# Continuous monitoring of eating and sleeping behaviors in the home environments of older adults: a case study demonstration

**DOI:** 10.3389/fpubh.2023.1277714

**Published:** 2024-01-12

**Authors:** Aditya Narayan, Margo Goncharova, Max Goncharov, Andrew Gostine, Nirav R. Shah, Robert M. Kaplan

**Affiliations:** ^1^Stanford University School of Medicine, Palo Alto, CA, United States; ^2^Artisight, Chicago, IL, United States; ^3^Northwestern Medicine, Chicago, IL, United States; ^4^Department of Medicine, Clinical Excellence Research Center, Stanford University, Palo Alto, CA, United States

**Keywords:** computer vision, activities of daily living, aging in place seniors, artifical inteligence, digital health

## Abstract

Accurate observation of patient functioning is necessary for rigorous clinical research and for improving the quality of patient care. However, clinic or laboratory environments systematically differ from the contexts of everyday life. Further, assessments that are completed in a single institutional session may not be generalizable. Here, we describe a computer vision methodology that measures human functioning continuously in the environments where patients live, sleep, and eat.

## Introduction

High quality health care delivery is dependent on objective patient observations. This process typically requires patients being present in a clinic or hospital. In the context of research studies, subjects are evaluated in precisely controlled laboratory environments. However, over 75 years ago, psychologist Egon Brunswik demonstrated that most outcomes of interest are modified or determined by a wide variety of contextual factors that may not be measured in the laboratory, clinic, or hospital setting (1). For instance, the level of social support, the accessibility and safety of the home environment, and daily routine variations can significantly impact an individual’s ability to perform Activities of Daily Living (ADLs) independently. In addition, the ambient environment including factors like air quality, noise levels, and access to green spaces can also play a pivotal role in affecting health outcomes. These factors are often intertwined with the individual’s health, cognitive capacity, and functioning but may not be captured adequately in a clinical or laboratory setting. Therefore, a holistic computer vision approach, such as the one with the pilot AI system, that captures these contextual factors in real-time within the individual’s natural living environment, is essential to provide a comprehensive understanding and support for older adults.

This reality has not changed since first described by Brunswik. Yet, what has changed is the capacity to study behavior in a wide range of natural environments and to understand the effects of context through the application of novel technologies (2). We now have methods that sample experiences, physical functioning, behaviors, and physiological responses from everyday environments and circumstances (3). New technologies, including cell phones, sensors, and wearables like smartwatches, have made it possible to move beyond self-reported outcomes, collect information and engage in research outside of sterile laboratory environments. However, an AI-based clinical decision support tool has the potential to offer a more nuanced and privacy-preserving method for monitoring older adults in their home environments. Specifically, computer vision can capture and analyze ADLs continuously in a real-world setting, providing richer insights into the functional health of older adults without intruding on their privacy.

One of the most appropriate applications of such technologies lies in the ability to monitor activities of daily living (ADLs). However, there are multiple challenges associated with measuring and monitoring ADLs in the home environment.

First, there is notable intra-and inter-individual variability in the performance of ADLs based on personal preferences, physiological capacity, and environmental context (4–8). Moreover, these factors may change over time in association with changes in health status. Accordingly, it is critical to paint a more comprehensive, longitudinal, and personalized picture of functional capacity. Second, there are numerous documented biases in ADL measurement including subjectivity, poor reliability, poor reproducibility, and low sensitivity to changes (4, 9, 10). Third, ADL observations are typically limited to a very small sample of behavior. We cannot assume that ADLs observed in a single clinic or laboratory visit will generalize to other times or environments. In sum, current ADL measurement approaches have limited power for predicting ADL-associated health outcomes or informing healthcare planning (11, 12). ADL monitoring that is capable of automatically capturing objective, continuous activity data in an ecologically valid approach can aid in the detection and prediction of clinically significant changes while facilitating supportive interventions to minimize adverse events.

Older adults, in particular, may benefit from flexible monitoring of ADLs. The number of older adults (65+) in the US is projected to grow rapidly in the coming decades, with a staggering five million or more individuals living with dementia in 2020 (3, 13). Often, seniors wish to remain in their homes to more effectively maintain their health-related quality of life (HRQoL) (14). This paper offers pilot data on an AI-based clinical decision support tool that utilizes computer vision to monitor older adults in their home environments.

## Methods

We used an AI/Machine Learning (AI/ML) tool for Computer Vision that utilizes a sensor as well as cameras with video and audio recording capacity, information processing, and an alerting mechanism. The algorithm applies machine learning to define behavioral norms in the home environment to ultimately provide explainable outcomes. This project was determined by Stanford Medicine IRB to not require IRB review given analysis neither constitutes human subjects research nor poses risk to patient privacy and well-being. Written consent was provided by the patient for installation of the technology.

The AI system evaluated in this study utilizes a combination of data collection, processing, and unsupervised machine learning (UML) to monitor ADLs and identify abnormal events. Initially, embedded sensors and cameras within the domestic environment are employed for continuous data acquisition across dimensions of time, location, video, and audio. This data is securely housed in an on-site processing unit, adhering to data privacy standards through an edge computing architecture. The processing phase engages UML to identify patterns within the collected data, establishing a baseline of normal daily activities for each individual. The system recognizes an individual through comparison of clothing colors, pace, posture, and other body movement characteristics and subsequently converts the image of the individual into a 3D stick figure, derived from a pose estimation model, with lines and points. Analyzes of behaviors are conducted using these stick figures represented in 3D, which are superimposed on pictures taken of the background every 30 s to create videos—those reviewing the video only see a stick figure engaging with their environment.

As the system continuously monitors real-time data, it compares the ongoing activities against the established baseline to detect deviations or abnormal activities. When such a deviation is detected, the algorithm evaluates and scores the event based on predefined criteria such as the duration spent in a specific zone and associated audio cues. For example, a person kneeling to pray—a posture that could be misinterpreted as a fall by other systems—is flagged for human review. Human operators can then review the flagged behavior, and upon recognizing it as a benign action, they can tag it accordingly. This tagged information is fed back into the AI system, improving its learning algorithm to correctly identify similar behaviors in the future. Moreover, the engagement with end-users and healthcare providers allows for a collective feedback loop, further refining the system’s understanding and recognition of a broader spectrum of behaviors. This iterative identification, tagging, and feedback mechanism enhances the AI system’s accuracy over time, reducing the likelihood of false alarms and ensuring a more precise and nuanced understanding of the monitored individuals’ actions. The process also facilitates a better adaptation to the diverse and personalized behaviors exhibited by different individuals, thereby improving the tool’s effectiveness in real-world, varied scenarios. Such events then trigger an alarm which may be sent to a team of human operators for verification and potential escalation to a designated caregiver/clinician. The feedback received from human operators and end-users may be utilized to refine the machine learning algorithm, enhancing the system’s accuracy and efficiency over time. Additionally, the AI system provides a dashboard for visualization of periodic summaries, real-time monitoring, and notifications of abnormal events. The generated data and insights are integrated into a clinical support system, assisting healthcare providers in making informed decisions regarding care planning and interventions.

The learning process of the AI system begins with a 1–2-week post-installation phase, where streaming video and audio data, securely stored on the local processing unit, are utilized to calibrate and establish the daily norms specific to an individual’s environment. This initial phase delineates the various zones and routine activities that the individual engages in daily. Following this, a dedicated ADL processor is deployed. This processor, fortified with trained models across the four key domains of duration, zone, video, and audio, analyzes ADL-related event clips. The data extracted from these clips is cross-referenced and correlated to activity zones, time durations, person postures, and related audios, which is then funneled into a trained machine learning model. This model iteratively adjusts a weighted coefficient for each domain parameter related to ADLs, enhancing the system’s precision over time. In time, the system undergoes further refinement to improve its reliability in determining the execution of particular ADLs. Installation and setup of the AI system involves placing sensors and cameras within the home environment at strategic locations to cover key areas where daily activities are likely to occur. A secure on-site processing unit setup is initiated to ensure data privacy while activating the system, with settings configured according to the user’s preferences and privacy considerations. During the data collection phase, the system continuously gathers data on the individual’s daily activities across four dimensions: time, location, video, and audio. Data privacy modes like privacy mode, utilizing “stick figures” or “pixelization” of human bodies, are activated based on the individual’s preference.

Periodic evaluation and adjustments are carried out to assess the system’s performance and the relevance of the data it provides in supporting healthcare planning. Necessary adjustments to the system settings, configurations, or placement of sensors and cameras are made to better suit the evolving needs of the individual. Further, education and support are provided to the individual, family members, caregivers, and clinicians to ensure a clear understanding of the system’s functionalities, benefits, and limitations. This fosters an environment of trust and informed utilization of the AI system. Over time, continuous improvement is pursued through the collection of feedback from all stakeholders to understand areas of improvement. System upgrades and improvements address any identified issues and keep the system updated with the latest technological advancements. Lastly, compliance and ethical considerations are upheld by ensuring adherence to legal and ethical guidelines, particularly around data privacy and security.

To gather relevant data, the AI system was deployed in a real-world setting, monitoring a cohort of older adults over a specified period. The AI system continuously captured and analyzed multidimensional data (spanning duration, zone, video, and audio domains) to recognize ADL patterns. The data were then aggregated and analyzed to assess the performance of the AI system concerning the ability to determine eating and sleeping behaviors. In our study, the primary outcome measures included the detection accuracy, sensitivity, and specificity of the system in identifying normal and abnormal ADL behaviors. These measures were derived from a systematic comparison between the AI-detected ADL events and the ground truth established through human annotation corroborated by the patient and their caregiver.

Initial analysis utilizing the Artisight software examined two key ADLs in a cohort of pilot patients. As proof of principle, we offer detailed results from the case of an 85-year-old patient diagnosed with vascular dementia who had recently experienced multiple falls without injury.

## Results

**Figure 1:**
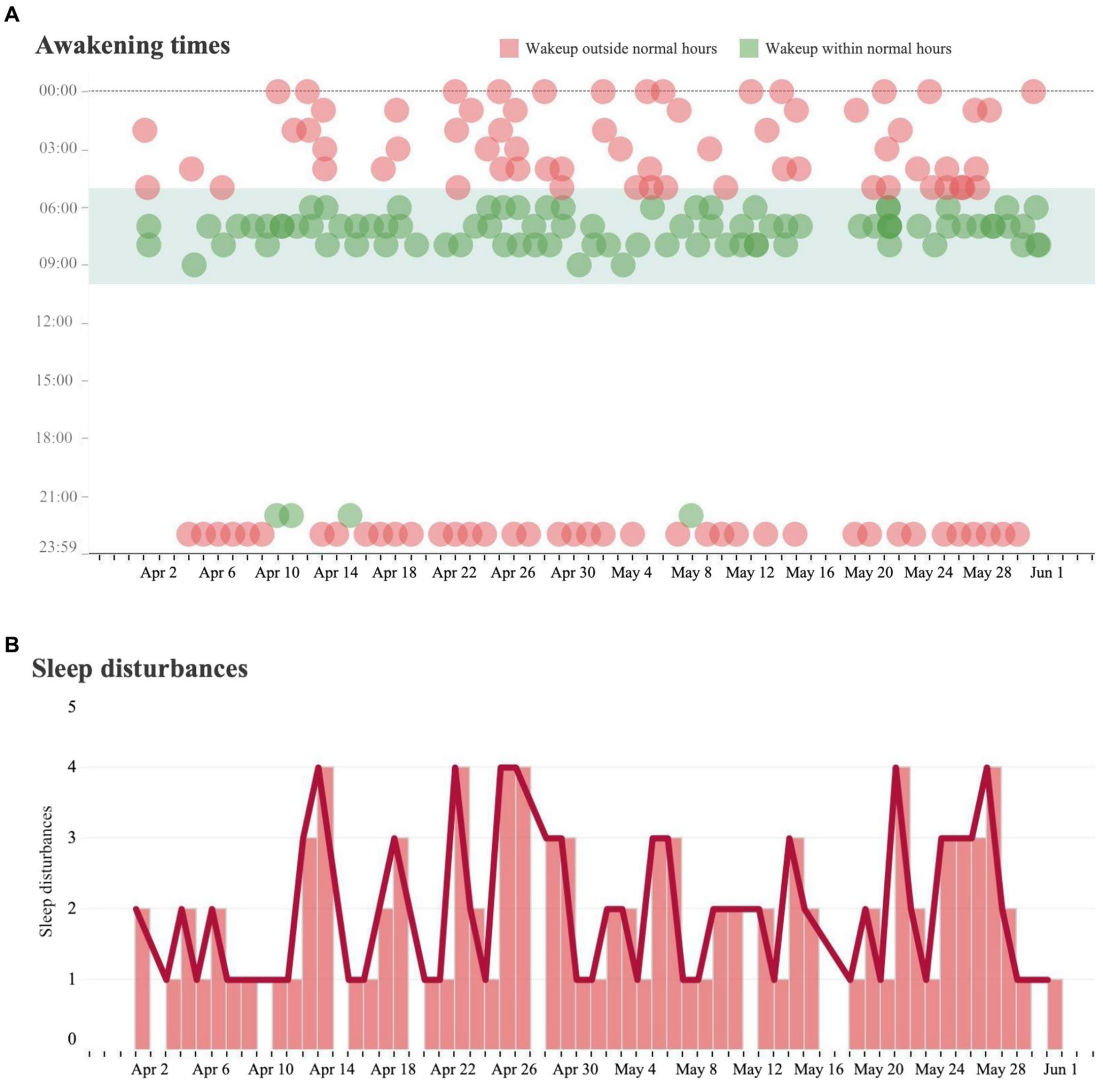
Sleeping analysis and sleeping disturbance analysis. **(A)** Each dot represents a measured instance of a patient waking from sleep over the course of the day from April 1 to May 31. If the patient awoke during a time that is outside of their baseline wakeup time, it was marked in red. If a patient woke up during a time within their baseline wakeup time, it was marked in green. **(B)** Trends in the number of sleep disturbances (waking outside of regular hours) per day over the same time period.

Results of initial analysis were based on data collected from January 1st, 2022, to July 1st, 2022. Normal sleeping patterns were defined by the caregiver and deviations from these patterns were identified as disturbances. In this case, disturbances were identified as awakening between the hours of 10 pm to 5 am (Figure 1). These events became increasingly common beginning in late March and early April.

**Figure 2:**
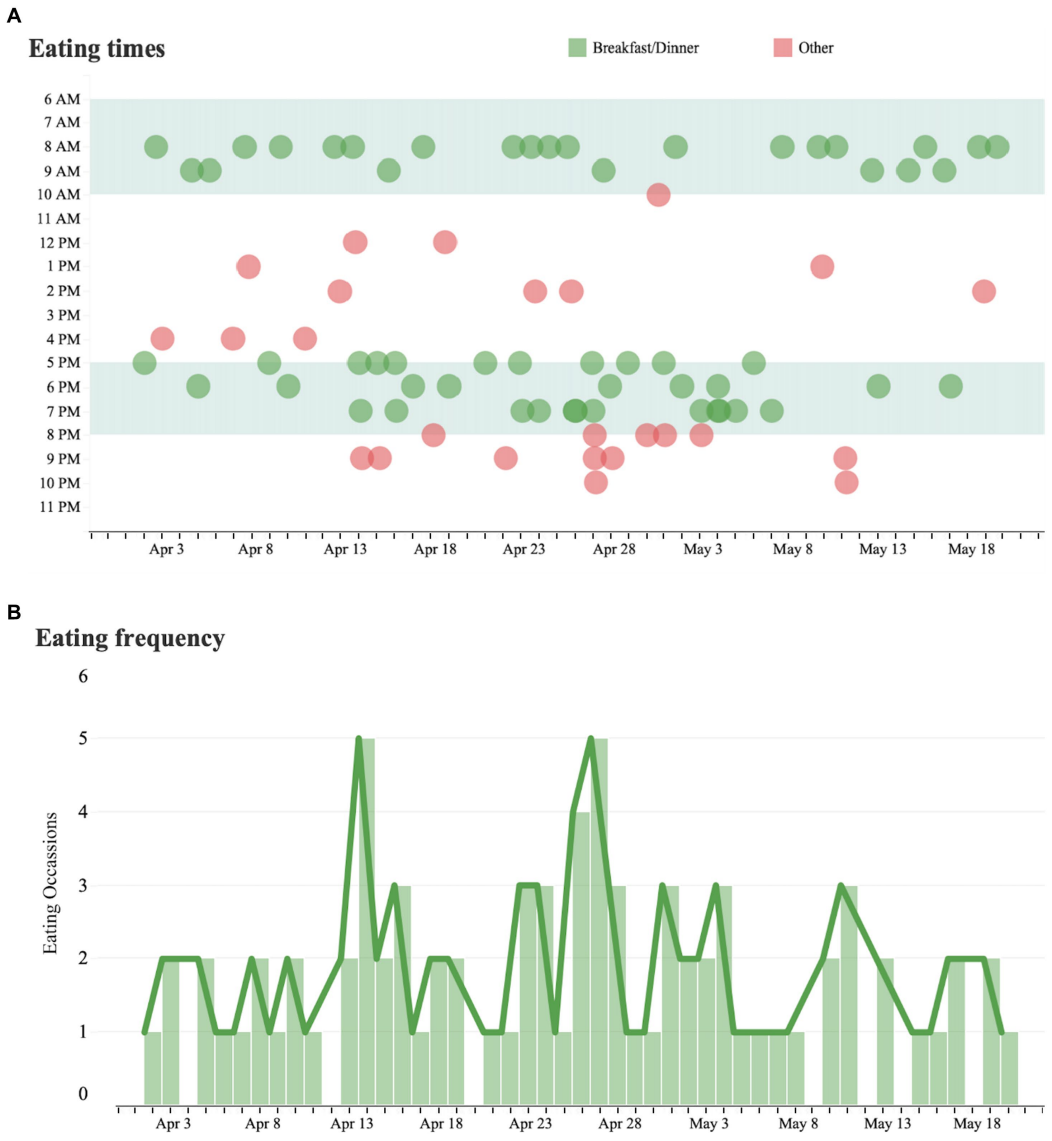
Eating time analysis and eating frequency. **(A)** Each dot represents a measured instance of a patient consuming a meal over the course of the day from April 1 to May 31. Eating times outside of the patient’s regular eating hours are marked in red and eating times within the patient’s regular hours are marked in green. **(B)** Trends in the number of eating occasions per day over the same time period.

Eating behavior was classified as the regular consumption of breakfast (meals consumed between 6 am to 10 am) or dinner (meals consumed between 5 pm to 8 pm), or as an alternative eating time (“other”). Eating at alternative times occurred regularly and became more common after late January (Figure 2).

## Discussion

A software tool for monitoring ADLs has the potential to provide real world obervations that are more detailed, complete, and free from many of the contextual biases common to the clinic or laboratory. Specifically, the ability to classify eating and sleeping behaviors allows for improved understanding of individual-level factors influencing patient’s health status as well as opportunities for health, structural, or social interventions.

The process of determining the ADLs used a multi-dimensional approach aimed at ensuring clinical relevancy. Initially, a review of existing literature was performed to establish fundamental ADLs crucial for upholding an independent living standard among the older adult population, particularly those at risk for AD/ADRD. This review illuminated common ADLs such as eating, bathing, toileting, dressing, transferring, and walking, which are often indicative of an individual’s functional status and overall quality of life. In tandem with the literature review, consultations with healthcare professionals specializing in geriatric care and long-term care settings were performed. The parameters were translated into learning models using a structured data annotation process to train the AI on key ADLs. Performance of the model was assessed through a pilot study with a small group of participants. To ensure internal consistency with these performance measures, the precision, recall, and F1 scores of the AI system in accurately measuring ADLs was measured. Additionally, iterative feedback loops between the system and human operators were used to iteratively refine the algorithm. In this pilot study, we focused on monitoring sleeping and eating behaviors as they are primarily driven by motion, allowing for a rigorous evaluation of this fundamental feature. This focus ensured the fidelity of the service in detecting and analyzing motion-based activities, providing a solid foundation for the system’s functionality before incorporating sound sensors in future evaluations. Although activities such as bathing and toileting are integral to assessing ADLs, their monitoring requires a careful and nuanced approach to ensure the privacy and dignity of individuals. Our AI system is designed to accommodate a wide range of ADLs, and the monitoring of more private activities like bathing and toileting will be addressed in future studies. This phased evaluation allows for the continuous refinement of privacy-preserving features, ensuring ethical and respectful monitoring of all ADLs in natural environments. Future evaluations will include an expanded list of ADLs.

Challenges with eating independently may lead to poor nutrition, dehydration, and the exacerbation of chronic health conditions. However, objective monitoring of food intake in contexts outside of the clinic remains an open challenge (15, 16). Similarly, there is minimal evidence in support of consumer technologies and wearables for the purpose of sleep monitoring (17). In contrast to sleep laboratories that typically evaluate patients on a single night in an unfamiliar environment, computer vision can offer assessments on multiple nights using data captured in the patient’s own bed.

The use of smartwatches and other wearables has provided a pathway to monitor some aspects of ADLs. However, these devices often require user interaction, charging, and may not capture the full spectrum of daily activities, especially those that are critical for evaluating the health and safety of older adults living at home. Computer vision stands apart in its ability to provide continuous, objective, and unobtrusive monitoring of ADLs in a real-world setting, thus providing clinicians with a more thorough understanding of an individual’s functional health and the necessary information to tailor interventions accordingly. Further, such accurate, comprehensive, and privacy-centric approaches to monitoring ADLs enhance the timely detection of abnormal and high-risk behaviors or critical events such as falls. This information is crucial for enabling older adults to live independently and safely at home. By identifying and addressing the unique challenges and preferences of older adults, computer vision methods may offer an important advance in home health monitoring, making a substantial stride towards personalized, effective, and dignified care for older adults.

Critically, the capacity of the technology for self-training may decrease upfront investment in technological and human capital. In training mode, an individual may perform an action. The algorithm tags the individual’s joints, angles, viewpoints, and timing associated with the action and from this, can generate video clips of the same action using variations of the aforementioned tags. This unsupervised learning approach minimizes upfront investment in training and, over time, false positive rates decrease as the system learns to identify various behaviors and subsequently apply it’s knowledge of identified behaviors in other contexts. The software tool may also be readily configured for human review of exceptions to effectively classify abnormal behaviors. In time, this may allow a single human operator to monitor a large number of video streams and focus attention primarily on abnormal behaviors.

A notable barrier to implementing AI-driven measures is the acceptability of the interventions. For providers, to address concerns about the transparency of algorithmic tools, the Artisight software shares the thresholds and parameters which are used to drive decision-making (18). Further, it displays results on a dashboard that allows clinicians to visualize data reports, summaries, order sets, and clinical guidelines to ultimately improve the clinical decision-making process. For patients, concerns regarding biometric data monitoring include alarm burden, privacy, and interference with daily life (19). By addressing patient privacy concerns, the tool offers the potential to increase monitoring interventions that may ultimately improve health.

Ultimately, the process of reliably modeling ADLs via the tool necessitated a robust data collection framework. We employed unsupervised machine learning initially to discern patterns and establish baselines from the multidimensional data captured which was stored with an eye towards privacy and data integrity. Further, to ensure acceptability by providers, iterative feedback led to the creation of a customizable dashboard that provides alerts, reminders, and summary reports enabled a practical translation of digital data into actionable insights for clinicians, caregivers, and family members. This dashboard, accessible via a secured platform, bridges digital data with real-world clinical care planning. The lessons learned from this project underscore the importance of an integrative, iterative, and collaborative approach in developing, deploying, and refining digital health technologies (20). Through the application of machine learning, human validation, real-world testing, and continuous feedback loops proved invaluable in advancing the AI system’s efficacy in monitoring ADLs and heralding meaningful alerts. As we move forward, we aim to further leverage these lessons to enhance the system’s sensitivity, specificity, patient-centricity, and overall utility in promoting better health outcomes for older adults.

## Limitations

Our conclusions must be considered in light of several limitiations. First, this is a pilot single case study used as proof of concept. Second, the algorithm has not been fully evaluated for accuracy and concordance with other ADL-monitoring tools. Third, the lack of observations of ADLs among older adults limits the potential generalizability of the study to older adults, individuals in different settings, and among much larger sample sizes. Moving forward, observations with a larger number of patients will be analyzed and reported.

## Conclusion

In the 20th century, Brunswik suggesed that acquiring large samples of individuals was less important than collecting observations of a few individuals in representative samples of situation. Progress in understanding the impact of behavioral settings has been slow because it has been difficult to observe individuals in their natural environments. Such research has been extremely demanding, expensive, and rarely feasible. New technologies and methodologies such as those presented in this paper may enable the collection of data in a wide range of natural environments. With informed consent, emerging tools are now capable of capturing information in natural physical and social environments. We are hopeful that these technologies will help make the science that Brunswik demanded more feasible.

## Data availability statement

The data supporting the conclusions of this article will be made available via the corresponding author.

## Ethics statement

The studies involving humans were approved by Stanford Medicine IRB to not require IRB review given analysis neither constitutes human subjects research nor poses risk to patient privacy and well-being. Written consent was provided by the patient for installation of the technology. The studies were conducted in accordance with the local legislation and institutional requirements. The participants provided their written informed consent to participate in this study. Written informed consent was obtained from the individual(s) for the publication of any potentially identifiable images or data included in this article.

## Author contributions

AN: Data curation, Investigation, Writing – original draft, Writing – review & editing. MarG: Data curation, Formal analysis, Software, Visualization, Writing – review & editing. MaxG: Data curation, Formal analysis, Methodology, Software, Writing – review & editing. AG: Conceptualization, Supervision, Writing – review & editing. NS: Conceptualization, Supervision, Writing – review & editing. RK: Conceptualization, Project administration, Supervision, Writing – original draft, Writing – review & editing.
